# Comorbidity of Type 2 Diabetes Mellitus and Depression: Clinical Evidence and Rationale for the Exacerbation of Cardiovascular Disease

**DOI:** 10.3389/fcvm.2022.861110

**Published:** 2022-03-10

**Authors:** Mengmeng Zhu, Yiwen Li, Binyu Luo, Jing Cui, Yanfei Liu, Yue Liu

**Affiliations:** ^1^National Clinical Research Centre for Chinese Medicine Cardiology, Xiyuan Hospital of China Academy of Chinese Medical Sciences, Beijing, China; ^2^Second Department of Geriatrics, Xiyuan Hospital of China Academy of Chinese Medical Sciences, Beijing, China

**Keywords:** type 2 diabetes mellitus (T2DM), depression, cardiovascular disease, clinical evidence, outlook

## Abstract

Depression is a common comorbidity of type 2 diabetes mellitus (T2DM). T2DM with comorbid depression increases the risk of cardiovascular events and death. Depression and T2DM and its macrovascular complications exhibited a two-way relationship. Regarding treatment, antidepressants can affect the development of T2DM and cardiovascular events, and hypoglycemic drugs can also affect the development of depression and cardiovascular events. The combination of these two types of medications may increase the risk of the first myocardial infarction. Herein, we review the latest research progress in the exacerbation of cardiovascular disease due to T2DM with comorbid depression and provide a rationale and an outlook for the prevention and treatment of cardiovascular disease in T2DM with comorbid depression.

## Introduction

According to the 2021 International Diabetes Federation data (1), the global incidence of diabetes is 10.5% and is expected to increase to 12.2% by 2,045, making it one of the fastest growing global major health events of the 21st century. Depression is a common comorbidity of type 2 diabetes mellitus (T2DM). The International Prevalence and Treatment of Diabetes and Depression (INTERPRET-DD) study (2) found that the incidence of major depressive disorder (MDD) in patients with T2DM was approximately 10.6%. Depression also has a high incidence in patients with cardiovascular disease (CVD) and is an important risk factor for CVD (3). Several clinical studies have shown that T2DM with comorbid depression increases the risk of CVD morbidity and mortality and is associated with secondary composite outcomes such as CVD mortality, myocardial infarction (MI), and cardiovascular surgery (4–6). The view that “diabetes is a cardiovascular disease risk equivalent” is now considered a consensus, and many studies have been conducted on the relationship and mechanism between the two. However, as an adverse psychological factor that affects T2DM and CVD, the effect of depression on T2DM with comorbid CVD remains to be determined.

In this mini review, the keywords “depression,” “depressive symptoms,” “depressive disorders,” “major depressive disorder,” “type 2 diabetes,” “type 2 diabetes mellitus,” “diabetes,” “cardiovascular disease,” “cardiovascular events,” “cardiovascular risk,” and “cardiovascular outcomes” were searched in the PubMed, MEDLINE, EMBASE, and Web of Science databases. Clinical studies on the effect of T2DM with comorbid depression on CVD published in the past 5 years (from January 1, 2017, to January 1, 2022) were reviewed, and the effects of T2DM with comorbid depression on cardiovascular disease were summarized, providing a clinical reference for future studies on the prevention and treatment of cardiovascular disease due to diabetes with comorbid depression.

## Comorbidity Characteristics

### T2DM With Comorbid Depression Increases the Risk of Cardiovascular Events and Mortality

T2DM with comorbid depression increases the risk of cardiovascular morbidity and mortality. A cohort study of 192,685 diabetic patients with or without depression showed that the risk of macrovascular complications, such as acute coronary syndrome and stroke, was 1.35-fold higher in diabetic patients with depression than in those without depression. This correlation was stronger in females than in males, and the severity of depression had different effects on the outcome of patients with T2DM. The correlation between recurrent MDD and macrovascular complications was stronger than that of isolated MDD and mild depression (7). A longitudinal study evaluating the two-way relationship between depression and macrovascular and microvascular complications of diabetes found that depression increases the risk of MI, coronary artery disease (CAD), congestive heart failure, cardiovascular surgery, and other composite T2DM-associated macrovascular events (8). A meta-analysis of 17 studies and over 1 million participants (5) found that depression was strongly associated with the risk of nonfatal and fatal CVD in patients with T2DM, providing evidence for the relationship between depression in patients with T2DM and cardiovascular events. Furthermore, depression was associated with an increased risk of major adverse cardiovascular events such as atherosclerotic cardiovascular disease (ASCVD), heart failure (HF), and MI in patients with early-onset T2DM. This association was not related to ethnic groups or age, and the correlation was stronger in females than in males (9).

These studies have underlined the association between T2DM with comorbid depression and composite cardiovascular events, and studies on the effects of T2DM with comorbid depression on coronary heart disease, MI, HF, and other diseases have also shown significant correlations. In patients with T2DM and comorbid depression, the risk of coronary heart disease and stroke increases by 36.8 and 32.9%, respectively, compared to patients with T2DM without depression (4). T2DM with comorbid depression increases the risk of MI compared to T2DM without depression, and persistent depression has a greater impact on the risk of MI than transient depression (10). This study not only demonstrated that T2DM with comorbid depression was associated with the development of MI but also confirmed that the course of depression was positively correlated with the risk of MI. Depression is prevalent in asymptomatic elderly patients with T2DM. In a study of the new-onset HF outcomes of 274 patients with asymptomatic T2DM followed up for a median period of 1.5 years, Cox regression analysis showed that comorbid depression increased the risk of HF in patients with asymptomatic T2DM by 2.5-fold. Furthermore, depression was a predictor of the incidence of HF in conjunction with poor glycemic control, left ventricular hypertrophy (LVH), and diastolic dysfunction (11).

In addition to predicting HF, depression can also be a predictor of cardiac ischemia in T2DM. In a 2-year follow-up study of asymptomatic T2DM patients with and without a history or symptoms of CAD, patients underwent myocardial perfusion single-photon emission computed tomography (MPS) and a depression and anxiety questionnaire. The results showed that depression and anxiety scores were predictors of cardiac ischemic events in patients with asymptomatic T2DM (12). However, due to the small number of new-onset cardiac ischemic events (*n* = 11) in the study, conclusions must still be verified through large-scale clinical trials. Depression can also be an independent predictor of all-cause mortality in T2DM. A large-scale prospective cohort study of 3,923 patients with T2DM in 56 primary health care centers (13) showed that the all-cause mortality in patients with depression was 1.4-fold that in patients without depression. The results of another large-scale cohort study in the T2DM population (14) also confirmed that depression increases the risk of mortality in patients with T2DM. In addition, depression can affect all-cause mortality in patients with prediabetes. Increased symptoms of depression increased all-cause mortality not only in patients with diabetes but also in patients with prediabetes; this correlation was stronger in patients with prediabetes (15).

T2DM with comorbid depression not only increases the risk of all-cause mortality but is also closely associated with increased cardiovascular mortality. The results of a meta-analysis (4) showed that patients with T2DM and comorbid depression had a 47.9% increase in cardiovascular mortality compared to patients with T2DM alone. A follow-up study of 1,495 patients over a median follow-up period of 7.7 years showed that patients with diabetes and depressive symptoms 1 year after enrollment were associated with increased eventual cardiovascular mortality (16).

### The Two-Way Relationship Between T2DM and Depression

Several clinical studies have shown that depression increases the risk of T2DM (17, 18) and that T2DM also increases the risk of depression (19); more severe depression is associated with a higher risk of T2DM (19). The 2020 guidelines of the Chinese Diabetes Society (20) clearly cited a possible two-way relationship between T2DM and depression and anxiety; T2DM exacerbates the development of depression and anxiety, and depression and anxiety increase the risk of T2DM. In addition, the guidelines also mentioned that specific populations of patients with gestational diabetes or postpartum diabetes have a higher risk of depression and anxiety. Gestational diabetes significantly increases the risk of postpartum depression and may be a risk factor for postpartum depression. Therefore, pregnant women with gestational diabetes must be screened for postpartum depression (21). Another prospective cohort study (22) found that women with prenatal depression were more likely to develop gestational diabetes than those without depressive symptoms. Thus, gestational diabetes mellitus and perinatal depression can also have a two-way relationship. Studies have shown that gestational diabetes increases the risk of future CVD in mothers (23). Currently, most studies have focused on the effect of gestational diabetes mellitus or depression alone on CVD or the effect of both on adverse pregnancy outcomes, and there have been relatively few studies on the effect of gestational diabetes mellitus with comorbid perinatal depression on CVD. Therefore, high-quality clinical studies are needed to investigate the effect of gestational diabetes mellitus with comorbid perinatal depression on cardiovascular outcomes in this specific population of pregnant women.

Depression increases the risk of macrovascular complications of T2DM. In addition, macrovascular complications of diabetes can also lead to an increased risk of depression (8). There are two-way effects between depression and macrovascular complications of diabetes, and the two are mutually influencing factors.

### Potential Mechanisms

The exact mechanism by which depression affects the risk of CVD in T2DM is unclear. Currently, it is believed to be associated with multiple pathways, such as neuroendocrine disorders and the vascular endothelial inflammatory response; these mechanisms are interrelated and not isolated (24, 25). Depression overstimulates the hypothalamic-pituitary-adrenal (HPA) axis, leading to excessive cortisol secretion, which aggravates vascular endothelial damage and promotes insulin resistance. In patients with depression, poor adherence to medications and poor lifestyle increase vulnerability of hyperglycemia and hyperinsulinemia, thus, promoting coagulopathy and fibrinolysis. Depression is also associated with elevated levels of inflammatory factors such as C-reactive protein (CRP), tumor necrosis factor-alpha (TNF-α), and interleukin 6 (IL-6). Hyperglycemia and insulin resistance in diabetes are known to promote endothelial dysfunction, inflammation, and abnormalities of coagulation and fibrinolysis, thus accelerating the development of atherosclerosis and CVD. Depression promotes these mechanisms to accelerate the development of CVD. Other studies have analyzed the association between mood disorders and diabetes and CVD (including hypertension and CAD) from a genetic perspective (26), finding that depression, diabetes, and CVD may have multiple shared potential pleiotropic genes, thus affecting multiple signaling pathways such as corticotropin-releasing hormone (CRH), adenosine monophosphate activated protein kinase (AMPK), and 5 hydroxytryptamine (5-HT) pathways.

## Treatment

### Pharmacological Treatments

#### Antidepressants

Patients with depression who received selective serotonin reuptake inhibitors (SSRIs), tricyclic antidepressants (TCAs), heterocyclic antidepressants, and other antidepressants have a significantly reduced the risk of developing T2DM (27). Moreover, long-term use of SSRIs (≥120 days) or TCAs (≥35 days) was associated with a lower risk of developing T2DM. Some researchers have studied the antidepressant dosage (28) and found that in diabetic patients with poor glycemic control with baseline hemoglobin A1c (HbA1c) of 9.29%, those treated with the optimal therapeutic dosage of antidepressants were more likely to achieve better glycemic control (HbA1c <7%) after 12 months. Therefore, optimizing antidepressant dosage in patients with T2DM may be beneficial for glycemic control.

Different classes of antidepressants have different effects on mortality and cardiovascular events in T2DM with comorbid depression. In a study of 36,276 patients with newly diagnosed diabetes and depression treated with different classes of antidepressants for 6 months, regular use of antidepressants was associated with a reduced risk of macrovascular complications and/or all-cause mortality (29). Another cohort study (30) also reached the same conclusion of reduced mortality in diabetic patients with comorbid depression with SSRIs and tricyclic/tetracyclic antidepressant treatment. Furthermore, the study also found that serotonin-norepinephrine reuptake inhibitors (SNRIs), norepinephrine-dopamine reuptake inhibitors, mirtazapine, and trazodone also significantly decreased the mortality risk, but the use of reversible inhibitors of monoamine oxidase-A (RIMA) increased mortality risk.

#### Oral Hypoglycemic Agents

Currently, numerous clinical studies have confirmed that sodium-glucose cotransporter 2 (SGLT2) inhibitors and glucagon-like peptide 1 (GLP-1) receptor agonists reduce the risk of CVD, and their clinical indications have also been included in treatment guidelines (31). However, few studies have investigated the effects of hypoglycemic agents on depression and no guidelines are available. Different classes of hypoglycemic agents have different effects on the risk of depression in patients with T2DM. The results of a retrospective cohort study of 40,214 adult patients with T2DM who were not treated or treated with single or combined oral hypoglycemic agents where depression was used as an outcome measure (32) showed that dipeptidyl peptidase 4 (DPP-4) inhibitors and SGLT-2 inhibitors were both significantly associated with a reduced risk of depression, while biguanides, sulfonylureas, α-glucosidase inhibitors, thiazolidinediones, and glinides did not show to be associated with a reduced risk of depression. Because only one patient in the study was treated with SGLT-2 inhibitors, and the study was a non-randomized retrospective study with many confounding factors, the conclusions must be further verified. There have also been different conclusions regarding the effects of metformin on depression. In a case-control study of 550 elderly diabetic patients in nine communities, Chen et al. (33) found that the risk of depression in patients treated with metformin was decreased compared with those not treated with metformin. However, this was a case-control study with a small sample size; hence, it cannot be concluded that metformin reduces depression in elderly patients with diabetes, but it only shows that metformin may be a protective factor for depression in elderly patients with diabetes. Danish researchers (34) tracked patients receiving antidiabetic drugs or insulin therapy between 2005 and 2015 through Danish population-based registers and found that continued use of metformin and combinations of drugs were associated with decreased rates of depression.

#### Antidepressants and Hypoglycemic Agents

In the last 5 years, studies on the effects of antidepressants combined with antidiabetic drugs on CVD remain lacking. However, in 2016, a Swedish study investigating the risk of first MI in patients undergoing antidiabetic and/or antidepressant treatment (35) found that the combination of antidepressants and antidiabetic drugs significantly increased the risk of first MI compared to drugs alone or without drugs. This risk was greater in middle-aged females (45–64 years) than in middle-aged males.

### Non-pharmacological Treatments

Non-drug therapies for diabetes with comorbid depression, such as psychosocial intervention and self-management, have proven to be effective. A randomized controlled trial found that a collaborative care model of behavioral activation and skills to support self-management, such as adherence to diet plans, exercise, medication, tobacco cessation, and follow-up visits with physicians, improved depressive symptoms and resulted in fewer cardiovascular events or hospitalizations than usual care (36). A meta-analysis of 31 randomized controlled studies (37) also indicated that psychosocial interventions could significantly improve depressive symptoms in patients with T2DM and depression, as well as improve glycemic control. Therefore, including psychosocial interventions in the management of T2DM may also be beneficial for glycemic control, but the impact on CVD outcomes may have a lack of evidence from large RCTs. In addition, another interesting study (38) suggest that rural residence may have a protective effect on veterans' mental health, indicating that the environment is also an important aspect of psychological influence, but the effect on T2DM is not yet known. In recent years, neuromodulation methods such as transcranial magnetic stimulation (TMS) (39, 40) and transcranial direct current stimulation (tDCS) (41) have also been proved to be effective treatments for depression. In the treatment of diabetes, deep repetitive TMS can promote weight loss in obesity and thus prevent T2DM (42), and tDCS treatment can improve the quality of life and physical function of patients with diabetic polyneuropathy (43). The results of a single-blind cross-over trial showed that double tDCS improved both glucose tolerance and stress axes activity in healthy men, so repetitive tDCS may be a promising non-pharmacological treatment option for improving glucose tolerance in patients with T2DM (44).

## Perspective

T2DM with comorbid depression has an increased risk of cardiovascular events and death. Depression and T2DM and its macrovascular complications influence each other and exhibit a two-way relationship. Depression is involved in and accelerates the development and progression of cardiovascular disease in diabetic patients. Therefore, it is particularly important to screen for depression in diabetic patients to provide further intervention or psychological treatment to reduce the risk of CVD. Several guidelines have recommended screening and intervention for depression in patients with diabetes (Table 1) (20, 45–50), but most of the guidelines lack uniform standards for populations requiring screening and associated tools and interventions.

**Table 1:** Guidelines for depression screening and interventions for patients with diabetes.

Guideline for the Prevention and Treatment of Type 2 Diabetes Mellitus in China (2020 edition) (20)	The mental status of patients with diabetes mellitus and depression and anxiety disorders should always be evaluated throughout the treatment. Early screening, evaluation, and monitoring of psychological status, especially in diabetic patients with a history of depression and anxiety, should emphasize emotional assessment when conditions change (e.g., development of complications) or when other psychosocial factors are present.	The collaborative care model can significantly improve depression and glycemic control in patients with diabetes and depression and reduce medical costs. Diabetic patients with depression should be referred to a psychiatrist with familiarity and knowledge of diabetes. Selective serotonin reuptake inhibitors and serotonin and norepinephrine reuptake inhibitors are often the first-line drugs of choice for diabetic patients with depression and anxiety and can improve glycemic control.
Standards of Medical Care in Diabetes—2022 (45)	Providers should consider the annual screening of all patients with diabetes, especially those with a self-reported history of depression, for depressive symptoms with age-appropriate depression screening measures, recognizing that further evaluation will be necessary for individuals who have a positive screening.	Referrals for the treatment of depression should be made to mental health providers who have experience using cognitive behavioral therapy, interpersonal therapy, or other evidence-based treatment approaches in conjunction with collaborative care with the patient's diabetes treatment team.
Precision Medicine in Diabetes: A Consensus Report from the American Diabetes Association (ADA) and the European Association for the Study of Diabetes (EASD) (46)	Providers must assess symptoms of depression using appropriate standardized and validated tools at the initial visit, at periodic intervals.	Psychological counseling can help patients understand and manage their emotional reactions to major events by developing a more optimistic outlook and more realistic, modulated, and adaptive emotional reactions.
Diabetes Self-management Education and Support in Adults with Type 2 Diabetes: A Consensus Report of the American Diabetes Association, the Association of Diabetes Care & Education Specialists, the Academy of Nutrition and Dietetics, the American Academy of Family Physicians, the American Academy of PAs, the American Association of Nurse Practitioners, and the American Pharmacists Association (47)	The identification of diabetes-related complications or other individual factors (e.g., psychosocial factors such as depression and anxiety) that may influence self-management should be considered a critical indicator of the need for DSMES that requires immediate attention and adequate resources.	Focused emotional support may be needed for depression. Additional mental health resources are generally required to address clinical depression.
Consensus statement of the American Association of Clinical Endocrinologists and American College of Endocrinology on the comprehensive type 2 diabetes management algorithm—2020 Executive Summary (48)	Healthcare professionals should assess the mood and psychological well-being of patients.	Healthcare professionals should refer patients with mood disorders to a mental healthcare facility.
Japanese Clinical Practice Guideline for Diabetes 2019 (49)	After at-risk patients with diabetes are screened for depression, systematically coordinated care is essential for both diabetes and depression.	An intervention that addresses both depressive symptoms and diabetes-related mental distress and anxiety is required to improve self-care ability and glycemic control in affected patients.
Diabetes Canada 2018 Clinical Practice Guidelines for the Prevention and Management of Diabetes in Canada: Diabetes and Mental Health (50)	Individuals with diabetes should be regularly screened for psychiatric disorders (e.g., depression) using validated self-report questionnaires or clinical interviews. Children and adolescents with diabetes should be screened for major depressive disorder and screened regularly for psychosocial difficulties, family distress, or mental health disorders.	Depression with diabetes should be referred to specialized mental healthcare professionals. Collaborative care by interprofessional teams should be provided for individuals with diabetes and depression. Antidepressant medications should be used to treat acute depression in people with diabetes and as a maintenance treatment to prevent recurrence of depression. Cognitive behavior therapy can be used to treat depression in individuals with depression alone or in combination with antidepressant medication.

Different classes of hypoglycemic agents have different effects on depression, and different classes of antidepressants have different effects on macrovascular complications and mortality in T2DM. In cases of T2DM with comorbid depression, there is no uniform conclusion on the impact of the simultaneous selection of hypoglycemic agents and antidepressants on the subsequent risk and prognosis of CVD. The effect of newly available hypoglycemic agents on depression is also a future research direction. The peroxisome proliferator-activated receptors (PPAR) pan-agonist Chiglitazar sodium (Bilessglu) was officially approved for the Chinese market as a novel hypoglycemic agent. Animal studies have shown that PPAR-δ is associated with depression-like behavior in a mouse model of chronic social defeat stress (CSDS) (51). Future clinical trials may determine whether Chiglitazar sodium can be used to improve depressive symptoms in diabetic patients. Due to the low side effects and low cost of neuromodulatory treatment, it can avoid the adverse reactions caused by the combination of multiple oral drugs, future studies on the clinical effects of T2DM with depression co-morbidities could also be conducted, thus enriching the options for its adjunctive therapies.

## Author Contributions

MZ and YiL formed the reference collection, conducted the reference analysis, and wrote the manuscript. YuL and YaL contributed to the topic conception, manuscript revision, and decision to submit for publication. JC and BL contributed to reference analysis and helped in the revision of the manuscript. All authors contributed to the article and approved the submitted version.

## Funding

This work was supported by the Outstanding Youth Foundation of National Natural Science Foundation (82022076) and the Special Project for Outstanding Young Talents of China Academy of Chinese Medical Sciences (ZZ15-YQ-017).

## Conflict of Interest

The authors declare that the research was conducted in the absence of any commercial or financial relationships that could be construed as a potential conflict of interest.

## Publisher's Note

All claims expressed in this article are solely those of the authors and do not necessarily represent those of their affiliated organizations, or those of the publisher, the editors and the reviewers. Any product that may be evaluated in this article, or claim that may be made by its manufacturer, is not guaranteed or endorsed by the publisher.
